# Advancing risk management in nuclear medicine diagnostic and therapy through incident-driven risk management tools^☆^

**DOI:** 10.1016/j.zemedi.2025.03.004

**Published:** 2025-05-20

**Authors:** Lidia Strigari, David Menichelli, Elisa Lodi Rizzini, Arber Golemi, Gian Mauro Sacchetti, Lucia Leva, Cristina Nanni, Paolo Castelucci, Stefano Fanti, Alessio Giuseppe Morganti, Roberta Matheoud

**Affiliations:** a Department of Medical Physics, IRCCS Azienda Ospedaliero-Universitaria di Bologna, Bologna, Italy; b IBA Dosimetry, Bahnhofstraße 5, 90592 Schwarzenbruck, Germany; c Radiation Oncology, IRCCS Azienda Ospedaliero-Universitaria di Bologna, Bologna, Italy; d Nuclear Medicine, IRCCS Azienda Ospedaliero-Universitaria di Bologna, Bologna, Italy; e Nuclear Medicine Department, University Hospital Maggiore della Carità, Novara, Italy; f Department of Medical and Surgical Sciences (DIMEC), Alma Mater Studiorum University of Bologna, Bologna, Italy; g Medical Physics Department, University Hospital Maggiore della Carità, Novara, Italy

**Keywords:** Radiation safety, Patient risk analysis, Nuclear medicine, Radioembolization, Radioligand therapy, Extravasation

## Abstract

Accidental or unintended exposures in nuclear medicine present significant risks, demanding proactive and systematic risk management strategies. This study explores the development and implementation of a novel software tool that integrates Failure Modes and Effects Analysis (FMEA) and Fault Tree Analysis (FTA) into a unified platform tailored for nuclear medicine. The tool addresses the complexities of risk assessment across diagnostic and therapeutic workflows, offering customizable templates and a streamlined process for identifying, prioritizing, and mitigating failure modes.

A multicenter study involving nuclear medicine departments of various sizes demonstrated the tool’s efficacy in standardizing risk analysis and enhancing interdisciplinary collaboration. Key scenarios, such as errors in radiopharmaceutical preparation and administration, were assessed, with rankings assigned based on a refined Risk Priority Number (RPN) system.

The results underscore the transformative potential of combining FMEA and FTA in nuclear medicine, addressing the limitations of standalone methodologies. This approach improves workflow efficiency and ensures a robust framework for patient safety. Future directions include expanding the tool’s applications, refining templates, and fostering a proactive culture of risk assessment. These advancements pave the way for safer, more efficient practices in nuclear medicine, benefiting patients and professionals alike.

## Introduction

European Council Directive 2013/59/EURATOM [1] requires that Member States ensure that (a) all reasonable measures are taken to minimize the probability and magnitude of accidental or unintended exposures of individuals subject to medical exposure, and (b) for radiotherapeutic practices the quality assurance program includes a study of the risk of accidental or unintended exposures.

Unintended exposures in the context of medical procedures are taken as meaning the exposure of a patient to a different radiation level than it was planned for the procedure carried out [2].

If the patient risk analysis in radiotherapy practice has been thoroughly explored and several reports, papers and guidelines have been published by international commissions and organizations [[3], [4], [5], [6], [7]], the risk analysis for nuclear medicine procedures has been studied only recently [2,8,9].

As far as nuclear medicine is concerned, unintended exposures are related to misadministration of radiopharmaceutical activities to patients for both diagnostic and therapeutic procedures. In this context, misadministration refers to the inadvertent administration of the radiopharmaceutical to an incorrect patient, the administration of an inappropriate radiopharmaceutical or an incorrect activity to the intended patient and the unwarranted performance of examinations on pregnant or lactating female patients [10]. Another form of misadministration involves utilizing an improperly administered activity that could result in significantly elevated absorbed exposure of non-target organs or undertreatment of tumor during therapeutic approaches. It is crucial to establish a local definition for incorrect activity. Generally, a deviation within ±5 % from the prescribed activity is considered acceptable in diagnostic applications [11,12].

A prospective and detailed analysis of the risk for each step in a nuclear medicine process is a proactive approach to risk management and sensitizes facilities to prevent failures associated with diagnostic/therapeutic processes before they are clinically implemented.

As a method of choice, many practitioners utilized the (process) failure modes and effects analysis, (P-)FMEA, which systematically identifies potential failure chains within a defined process [[13], [14], [15]]. The FMEA allows the identification of many singular failure modes, i.e., failure modes immediately causing process failures. The FMEA usually relies on a criticality analysis in which experts evaluate these failure chains quantitatively. Based on these evaluations, a risk priority number (RPN) is obtained as a surrogate for ranking and prioritizing subsequent actions to prevent potential failures. However, the method cannot model dynamics between failure modes even though it is known that failure modes indeed follow error pathways before they negatively affect a scenario [16]. In their report N. 181 [13], the European Commission stated that fault tree analysis (FTA), is suitable for more in-depth assessments using input data previously obtained through FMEA. Then, in 2016, the AAPM TG-100 report demonstrated in detail how both FMEA and FTA could be combined to describe better the risk profile of a radiotherapy treatment process [14]. Spreadsheets are commonly used to list the treatment process and perform FMEA, while visual tools such as flowcharts and fault trees are usually unsupported.

Consequently, several software tools are needed to describe the relationships between process steps, decision paths, failure modes and their dependencies, and measures to be taken to minimize the onset of the risk. These relationships should rely on a single dataset and tool to maintain data integrity and version control. In addition, a potentially emerging problem is the overarching prioritization of failure modes from different individual risk assessments, e.g., concerning different procedures and scenarios. The question arises about the overarching ranking if all failure modes were compiled in a single combined list. The setup of the entire risk analysis for each process in a nuclear medicine department is cumbersome and requires great efforts from the different professionals involved. This is particularly relevant when considering that after all, the total hospital resources are limited, and all the required actions in the process described above are drawn from these resources. Therefore, a software tool that combines a formalized approach to risk assessment and the possibility to personalize nuclear medicine department specific scenarios would deeply assist the professionals in their duty. The feasibility of commercially available software for radiation therapy has been evaluated for use in Nuclear Medicine, and a dedicated template for Nuclear Medicine has been developed. To the best of the authors' knowledge, this software enables a formalized approach to risk assessment and combines FMEA and FTA was developed to incorporate nuclear medicine scenarios. Second, the tool was commissioned for clinical use and validated using existing risk assessments in a multicentric setting. Third, a proposed method was tested that gives different weights to the criticality (the RPNs) of failure modes from different workflows and calculates the overarching ranking considering small, medium, and large nuclear medicine departments. Finally, the list of risk analysis templates currently available for the software was implemented, considering the nuclear medicine diagnostic or therapeutic scenarios. Templates were based on data from the cited literature, which have been simplified, generalized, and, if necessary, integrated based on the author’s experience.

## Methods and materials

### Software application

MyQA® PROactive (Ion Beam Application Dosimetry, v. 2.12.0.0) [16], is a web database application managed by the SQLite database engine (public domain). Risk analysis experts could access the web interface with any available workstation since no client-wise installation was required. As detailed in reference [17], three complementary risk assessment tools were implemented: FMEA, FTA, and Failure Modes and Effects Summary (FMES), briefly summarized in the following sections.

#### FMEA

FMEA is a systematic method that identifies potential failure chains whereby a failure chain consists of a failure cause, failure mode, and failure effect [14]. Failure modes are manners in which process failures occur. Traditionally, a 'bottom-up' approach is followed, where detailed processes are examined sequentially, and the consequences of failures at lower levels are inferred for higher levels. In contrast, the 'top-down' approach involves identifying process functions first and then investigating functions contributing to pre-identified top events. After the analysis, failure chains are quantitatively evaluated to determine criticality, considering occurrence (O), detection (D), and severity (S) of their respective effects.

The RPN, i.e., the product of S, O, and D, was used as a criticality surrogate, as presented in the AAPM TG-100 task report (see Table II in [14]), i.e., as a ten-step rating system with scores ranging between 1 and 10. Finally, barriers are identified to optimize the process, beginning with the highest-rated failure modes. Moreover, barriers can be either proactive or reactive. Whereas proactive barriers aim to prevent failure mode, reactive barriers are supposed to detect failures in case they occur before harm is generated. In other words, proactive barriers reduce O, and reactive barriers decrease D. This mechanism was achieved by introducing a reduction factor P_miss_ that estimates the effectiveness of the respective barrier. By assuming barriers i to be independent of each other, the residual (optimized) RPN could be obtained with RPNout.

The probability that a failure mode occurs is:

(1) $$P_{\mathrm{occ}}=P_{\mathrm{occ},i}∙\prod_{\begin{array}{c} \mathrm{proactive}\\ barriers \end{array}}p_{\mathrm{miss}}$$

where P_occ,i_ = P_occ,i_(O_i_) is the occurrence rate corresponding to the initial evaluation of occurrence O_i_. The new O value with proactive barriers in place can be determined since P_occ_ is a monotonically increasing function of O.

The probability that the failure mode remains undetected and generates the effect on a patient (adverse event) is:

(2) $$\left(1-P_{\det}\right)=P_{\mathrm{miss},i}\prod_{\begin{array}{c} \mathrm{reactive}\\ barriers \end{array}}p_{\mathrm{miss}}$$

where P_miss,i_ = P_miss,i_(D_i_) is the probability corresponding to the initial evaluation of detectability D_i_. The new D values with reactive barriers in place can be determined because (1-Pdet) is a monotonically increasing function of D. Then new values can be used to update the RPN:

(3) $$\mathrm{RP}N_{i}={S∙O}_{i}∙D_{i}$$

#### FTA

FTA is a 'top-down' Boolean logic tool that describes fault events quantitatively or qualitatively, building an error pathway through logical operations that lead to a top event. A graphical tree is developed whereby the top event is the pre-identified event of interest and is positioned on the top of the fault tree. Starting from the top, its possible branches are developed further downstream to identify associated intermediate and basic events of failure that contribute to the top event. Events may occur in failure modes or failed barriers. Basic events are such events not developed any further as they may be the cause of failure or beyond the system boundaries.

#### Failure modes and effects summary (FMES)

FMES is a method for grouping failure modes of an FMEA that produces the same potential effect to reduce the data input for higher-level FMEAs or FTAs [16,17]. First, all failure modes are analyzed, and their potential effects on the highest system level are categorized.

Then, all failure modes in the FMEA that cause the same effect are summarized as one failure mode in the FMES, with the failure modes of the FMEA then becoming its causes.

**Fig. 1:**
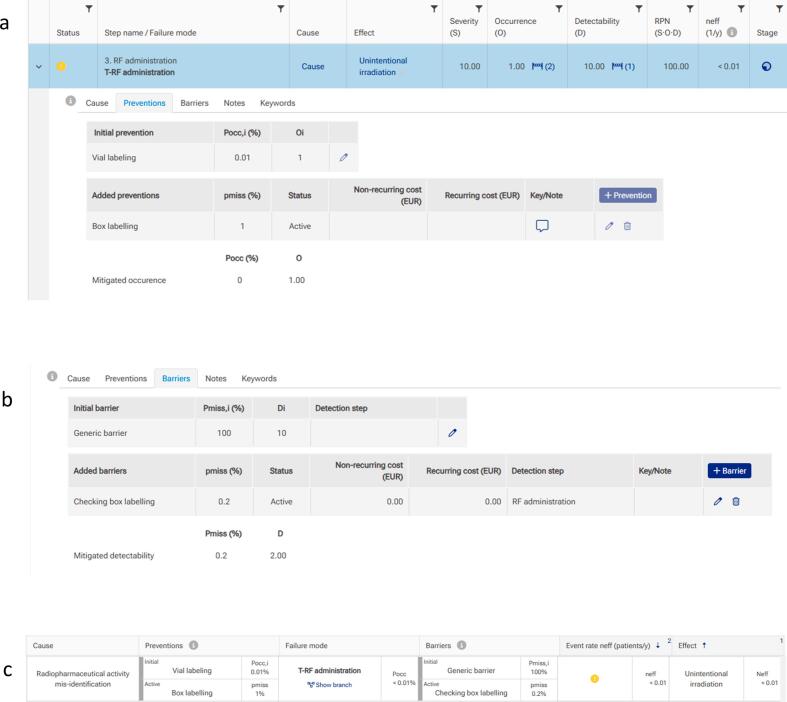
Example of steps for assessing the risk of incorrect vial administration during 90Y-microsphere radioembolization therapy: a) initial prevention is ‘*vial labelling’* with P_occ,i_ = 0.01(%) and O_i_ = 1, added prevention is ‘*box labelling’* with P_miss_ = 1 % and O = 1; b) adding the barrier ‘Checking box labelling’ decreases P_miss_ to 0.2 % and D = 2.00; c) added prevention and barrier reduce unintentional irradiation to N_eff_ < 0.01.

Risk assessment data were converted between FMEA and FTA by applying the FMES algorithm, as depicted in Fig. 1. The approach is novel, and a patent request has been deposited [17]. Failure modes of the FMEA and the fault events of the FTA were treated the same way to this end. This allowed for the results obtained by FMEA and FTA to be represented as a table and a fault tree. Combining both methods, the N_eff_ rate at which a top event occurs and remains undetected could be calculated. Each branch is described by a single failure caused by a failure mode/fault event and barriers are connected by ‘AND’ gates. Therefore, for each failure *j*

(4) $$N_{\mathrm{eff},j}=P_{\mathrm{occ}}^{j}\times (1{-P}_{\det}^{j})$$

Using OR gates to join the branches, the respective event rates on the next higher level were given by summing the N_eff,j_. The sum of the N_eff,j_ can be converted from event rates to events per year by multiplying it by the absolute patient throughput per year. As an additional way of prioritizing, only branches exceeding a particular threshold for severity could be displayed, and those contributing higher shares to the top event further analyzed.

### Questionnaire implementation and investigated cases

The multicenter study to evaluate the clinical risk in Nuclear Medicine involved two tertiary care centers, IRCCS AOU Bologna (Italy) and the University Hospital Maggiore della Carità – Novara (Italy), including N.1 Conventional NM diagnostic service 'small' (1300 patients/year) N.1 Conventional NM and 'medium' PET diagnostic service (8000 pcs/y) N.1 Conventional NM and 'large' PET diagnostic service (14000 patients/year) N.1 'small' NM therapy service (100 patients/year) and N.1 'medium' NM therapy service (400 patients/year)

The first step to analyzing hypothetical clinical risk scenarios involves identifying the phases of the patient's workflow within the Nuclear Medicine department, delivering diagnostic examinations or therapeutic procedures. The analysis comprised all the steps for nuclear medicine diagnostic: patient admittance, radiopharmaceutical /activity preparation, medical history, radiopharmaceutical administration, image acquisition for diagnostic examination, image processing, patient discharge, archiving/reporting.

Two preliminary questionnaires (Table S1a and S1b of Supplemental materials) were prepared for a more accurate assessment of the clinical risk, which was administered to the various professional figures to complete: nuclear medicine physicians, nurses, administrative, technicians, radio pharmacists, and medical physicists.

Data collected included, for example, Participant Center, Indicative number of patients/year, Professional, Diagnostic or therapeutic scenario, Hypothesized event, Expected cases (Number/year), Severity, Occurrence, chance of prevention, Detectability, and Cause of failure to be classified among organizational, procedural, technical, training, Informatic technology.

Examples of examined scenarios included: errors in radiopharmaceutical preparation/labelling, radiopharmaceutical administration to pregnant patients, Interruption of administration for clinical reasons, Incorrect attribution of examination, administration different from that planned.

#### Commissioning the software

With the intent to use the 'top-down' FMEA approach for future risk assessments [3], the software was commissioned for clinical use according to ad hoc developed steps for nuclear medicine diagnostics and therapy. Firstly, a standard process map was used for almost all future risk assessments.

The process map contained several local steps to reduce overlap between future scenarios applicable to nuclear medicine risk assessments. Secondly, functions and associated failures of the general nuclear medicine process were established. The process functions were identified by analyzing the underlying purpose of each process step. By accurately and concisely describing the process functions, process failures could be deduced by negating these functions. This step was undertaken to establish the same wording for standard events. Furthermore, using particular functions to create subsets of the process map was expected to help identify more relevant failure modes.

Thirdly, the rating system was adjusted according to the established procedure of our departments in a multicentric context. Instead of rating the occurrence probabilities, frequencies, such as failures per unit of time, were preferred. The severity parameter was also adjusted to our needs and associated with top events known for nuclear medicine therapy from the literature [18,19].

Based on these maps, all the distinct processes were identified, including scenarios potentially affecting the administration of radioactive drugs or devices of patients negatively. Each step of the scenarios was fully analyzed. In addition, the parameters of the rating system were adjusted to institution-specific needs.

Lastly, with the help of FMES the failure modes from two existing risk studies were analyzed to identify further top events. Again, this ensures consistent wording of failure effects. The top events were then used for FTA and placed on top of the process failures described above.

As described in the following sections, the tool was implemented for nuclear medicine diagnostic and therapy. Validation was considered successful when performing and continuing the risk assessment was feasible.

#### Analyzing existing risks

The Risk Priority Numbers (RPNs) were calculated and analyzed based on the scores provided by the involved professionals. Local process maps were aligned with a newly standardized process map to ensure consistency across workflows. Failure effects were redefined to maintain coherence with the identified process failures and associated top events. Barriers were categorized as either proactive or reactive, with their probability of failure (P_miss_) determined by comparing initial and residual occurrence rates. Finally, failure modes were ranked according to their criticality and sorted in descending order to prioritize risk mitigation efforts effectively.

## Results

### Software implementation for NM applications

A dedicated online risk assessment tool for nuclear medicine diagnostics and therapy was developed using a combined FMEA-FTA approach. The workflow was designed to systematically guide the user through all steps of the risk assessment. The first step of the workflow required the user to create the risk analysis under a rating system selection. Subsequently, a process map was required, described in the tool by a table or flowchart. The actual failure and risk analysis could be carried out at this point. Creating a failure mode always required the specification of a failure cause and effect and a rating of the parameters S, O, and D, resulting in an initial RPN_in_. This value, which refers to the worst-case scenario, could be reduced by adding either proactive or reactive barriers, i.e., introducing a reduction factor P_miss_ as shown in Fig.1.

**Fig. 2:**
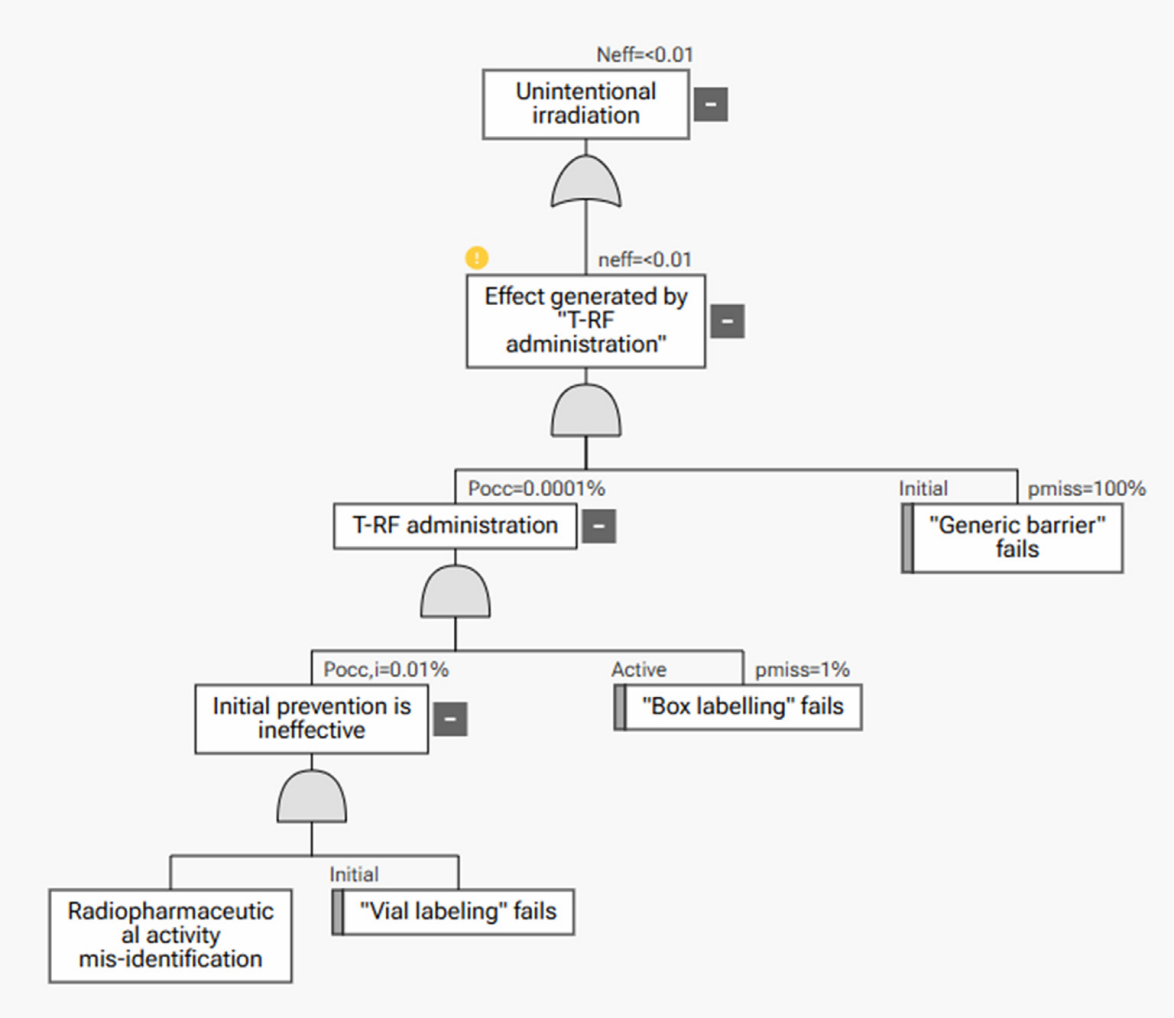
FTA analysis of the scenario of administering two vials with ^90^Y microsphere activity in an incorrect order during a radioembolization angiographic procedure.

As an alternative, the user can always set the initial values of occurrence probability and probability of not detecting a failure. After the creation of a failure mode, the tool automatically generated the equivalent fault tree representation by applying FMES (the failure cause became the basic event, the failure mode became the fault event, and the failure effect became the top event, as shown in Fig. 2). Moreover, all failure modes resulting in the same top event became the branches of a single fault tree. The workflow of the software and an exemplary fault tree consisting of two failure modes can be found in the Supplementary Materials Fig. S1. The myQA PROactive tool was commissioned for clinical use with the intent of a combined FMEA-FTA approach in general and a 'top-down' FMEA approach. To this end, the process map was adopted and analyzed. As for EBRT, the severity of the same event (rebooking of the examination) was scored differently by different professionals, particularly physicians and technicians. In these cases, a weighted score was adopted for analysis.

### Investigated scenarios

We collected a total of 7 steps, and 13 failure modes were hypothesized for diagnostic scenarios (including *Contamination of the gamma camera during a diagnostic procedure*, *Incorrect syringe identification*, or *incorrect activity administration – vial exchange during a therapeutic administration*, see supplementary materials **Table S2)**. The Severity score ranged from 1 to 10, the Occurrence was 1 or 2, while the Detectability ranged from 1 to 4. The RPN ranged from 1 to 20.

We collected a total of 8 steps, and 20 failure modes were hypothesized for the therapeutic scenarios, see supplementary materials **Table S3**. The Severity score ranged from 1 to 10, the Occurrence was 1 or 7, while the Detectability ranged from 1 to 10. The RPN ranged from 36 to 324.

The analyses of the risk related to extravasation of the radiopharmaceutical and vial exchange during a therapeutic procedure are reported in the following. The assumed use case concerns administering ^90^Y microspheres to a liver cancer patient as a representative therapeutic scenario (Fig. 1 and Table 1). This procedure is usually performed in one of the nuclear medicine department centers and represents the scenario among those with the highest risks for the patient. Often, the treatment approach can be based on multiple administrations of ^90^Y microspheres to the tumor feed, each of which may have a different activity chosen based on tumor characteristics, namely volume and perfusion. Usually, ^90^Y microsphere activities are prepared in single vials, each placed in a plexiglass box to reduce personnel irradiation during the procedure. The assumed use case relies on administering the activity of two vials having different 90Y microsphere activities, in an incorrect order during a radioembolization angiographic procedure. The proper labeling of the two vials (activity value, date/time of calibration, order of injection) is the initial prevention measure that can reduce the probability of exchanging the vials containing the activity to be injected into the hepatic arteries (i.e., bad administration). An additional prevention is the external box labelling. For this effect, the initial occurrence probability P_occ_ = 0.01 %, a P_miss_ = 1, and in case of administration had a potential occurrence O = 1. Potentially, this event could lead to a severity of 10, corresponding to a very wrong dose, absorbed dose distribution, and location of volume (Fig. 1a).

**Table 1:** Failure modes for a therapeutic NM scenario according to the initial Severity (S_in_), Occurrence (O_in_), Detectability (D_in_), RPN_in_.

Step/ Substep name	Cause of failure	Failure Mode	Initial Preventions	Initial Barriers	Severity, initial	Occurrence, initial	Detectability, initial	RPN_in_	Effect
Radiopharmaceutical Administration/ Device application	Lack of attention; lack of experience, time pressure	Vial with incorrect activity was handled to the physician	Consultation between doctor and nursing staff	Detected by chance	5	4	7	140	Temporary adverse effect
Radiopharmaceutical Administration/ Device application	Incorrect application of procedures; lack of experience, lack of attention	Radioembolization	Independent check by other staff members; flux evaluation by radiologist	Detected by chance	6	2	5	60	Temporary adverse effect

Adding the checking of the external box labelling (added barrier) could decrease the P_miss_ to 0.2 % and increase the event detectability D_i_ = 2 (Fig. 1b). This barrier will reduce the initial P_occ_ to 0.0001%, thus reducing the event rate to a N_eff_ < 0.01 (Fig. 1c). Based on the analysis of one of the therapeutic scenarios, incorrect administration can be prevented by labelling the vials with the different activities of Y90 (glass or resin) microspheres to be administered during the angiographic procedure, thus increasing the event detectability and reducing the probability of non-identifying the incorrect vial.

FTA representation of the above scenario is reported in Fig. 2.

Using FMES, Table 1 reports the highest-level failure effects of the possible failure modes for therapeutic applications. A many-to-many relationship exists between the process failures and top events, i.e., a particular process failure can result in more than one top event. The initial ranks obtained using the RPN and risk matrix were compared for plausibility.

## Discussion

The findings of this study emphasize the critical need to address errors in nuclear medicine in diagnostic [1] and particularly in therapeutic applications, where mistakes during treatment delivery or planning can lead to significant clinical consequences such as under-treatment or overexposure [8,21,22]. These risks highlight the importance of implementing accurate dose calculation protocols alongside robust quality assurance mechanisms to ensure optimal treatment outcomes.

The development and adoption of web-based notification systems, such as SINMED and SAFRON, offer significant opportunities to improve incident reporting in nuclear medicine. SINMED, with its user-friendly HTML5 interfaces integrated into platforms like Google Forms, facilitates real-time documentation and sharing of incidents. Similarly, SAFRON provides a centralized platform for global radiological incident reporting and analysis [7]. Expanding these systems to include nuclear medicine-specific incidents, particularly those related to therapy, could standardize safety protocols across institutions and foster international collaboration [19,20,21]. Such integration would enhance the collective understanding of risks and the development of effective mitigation strategies. One persistent challenge is harmonizing risk prioritization across diverse workflows and institutional practices. To enhance patient safety, an FMEA analysis was conducted to integrate a new microsphere product into existing 90Y microsphere program [18,22]. With the release of new guidelines [[23], [24], [25]], the previous FMEA assessment proved to be an effective foundation for future safety improvements. The Nuclear Medicine and Medical Physics department staff may evaluate and weigh risks differently, influenced by local protocols, available resources, and professional perspectives.

As demonstrated in this study, the combined application of FMEA and FTA within a unified tool presents a robust solution for addressing these disparities by providing a comprehensive and standardized approach to ranking and mitigating risks among nuclear medicine departments. In addition, the integration of FMEA and FTA within a single software platform marks a significant advancement in risk management practices. While FMEA provides granular insights into individual failure modes and their impacts, FTA complements it by modeling interdependencies among these failures, enabling a holistic view of error pathways. This dual-method approach overcomes the limitations of standalone methodologies and facilitates a deeper understanding of systemic risks and their mitigation. This innovation can potentially set new standards in the risk management of nuclear medicine processes.

By simplifying workflows and standardizing data formats, tools like myQA PROactive encourage meaningful collaboration among nuclear medicine professionals, including physicians, medical physicists, radiopharmacists, and technicians. This approach fosters a shared understanding of risks and ensures coordinated, multidisciplinary efforts to prioritize and mitigate failures. Enhanced collaboration can lead to more effective implementation of safety measures and improve overall patient care quality. Moreover, such an approach allows for the schematic description of the risk scenario considered, thus helping the professionals to add preventions and barriers in an easy and intuitive way.

The tool's flexibility and adaptability to different department sizes and clinical scenarios highlight its potential for scalability. Its customizable templates and intuitive interface make it a practical solution for diverse settings, extending its relevance beyond nuclear medicine into other domains where complex risk analyses are necessary. Broader adoption of such tools could drive improvements in risk management across healthcare systems, promoting consistency, efficiency, and safety.

While comprehensive risk assessment tools offer significant benefits, their implementation demands considerable resources, including time and skilled personnel. Hospital resource constraints necessitate a balanced approach, ensuring that risk management activities are both effective and sustainable. Tools like myQA PROactive, which combine multiple methodologies, streamline processes, and automate prioritization, can alleviate the resource burden, enabling facilities to maintain a high standard of safety without compromising operational efficiency.

## Conclusion

This study demonstrates the feasibility and value of integrating FMEA and FTA within a dedicated software platform to advance risk management in nuclear medicine. The tool has proven its ability to enhance safety, streamline workflows, and foster interdisciplinary collaboration. By offering a standardized framework for analyzing and prioritizing risks, the tool provides a critical resource for nuclear medicine departments striving to improve patient care and optimize resource utilization.

Future efforts should focus on expanding the tool’s applications to accommodate evolving technologies and workflows, refining the templates based on user feedback, and promoting a culture of proactive risk assessment. Strengthening partnerships with international organizations to harmonize incident reporting systems and encourage global collaboration will further enhance the safety and quality of nuclear medicine practices. These initiatives represent crucial steps toward reducing unintended exposures and ensuring the highest standards of patient safety in this critical field.

## CRediT authorship contribution statement

**Lidia Strigari:** Writing – review & editing, Writing – original draft, Validation, Methodology, Investigation, Conceptualization. **David Menichelli:** Writing – review & editing, Validation, Software, Investigation. **Elisa Lodi Rizzini:** Writing – original draft, Investigation, Data curation. **Arber Golemi:** Writing – original draft, Investigation, Data curation. **Gian Mauro Sacchetti:** Investigation, Data curation. **Lucia Leva:** Investigation, Data curation. **Cristina Nanni:** Investigation, Data curation. **Paolo Castelucci:** Investigation, Data curation. **Stefano Fanti:** Investigation, Data curation. **Alessio Giuseppe Morganti:** Investigation, Data curation. **Roberta Matheoud:** Writing – review & editing, Writing – original draft, Validation, Methodology.

## Declaration of competing interest

The authors declare that they have no known competing financial interests or personal relationships that could have appeared to influence the work reported in this paper.

## Appendix A Supplementary material

Supplementary Data 1 Failure modes for specific diagnostic and therapeutic scenarios considered in the paper.

## References

[1] European Council. Council Directive 2013/59/Euratom of 5 December 2013 laying down basic safety standards for protection against the dangers arising from exposure to ionising radiation. Official Journal of the European Union, No. L13; 2014. p. 73. 10.7417/CT.2024.5127 39400087

[2] Marengo M. (2021). Radiation safety and accidental radiation exposures in nuclear medicine. Semin Nucl Med.

[3] ICRP Publication 112 Preventing Accidental Exposures from New External Beam Radiation Therapy Technologies, International Commission in Radiation Protection 2009 Ann. ICRP 39 (4). 10.1016/j.icrp.2010.02.002 20478472

[4] Radiotherapy risk profile, Technical Manual, World Health Organization; 2008.

[5] IAEA (International Atomic Energy Agency) 2018 Radiation Protection and Safety in Medical Uses of Ionizing Radiation: Specific Safety Guide No. SSG-46. Available at: http://www.iaea.org/publications/11102/radiation-protection-and-safety-in-medical-uses-of-ionizing-radiation.

[6] Tramacere F., Sardaro A., Arcangeli S. (2021). Safety culture to improve accidental event reporting in radiotherapy. J Radiat Prot.

[7] IAEA. SAFRON: Safety and learning system for radiotherapy. Safety in Radiation Oncology. Available from: https://www.iaea.org/resources/rpop/resources/databases-and-learning-systems/safron. [Accessed 30 June 2021].

[8] Maughan N.M. (2022). Failure modes and effects analysis of pediatric I-131 MIBG therapy: program design and potential pitfalls. Paediatr Blood Cancer.

[9] Younge K.C. (2016). Failure mode and effects analysis in a dual-product microsphere brachytherapy environment, 2016. Pract Radiat Oncol.

[10] IAEA. Misadministrations in diagnostic nuclear medicine. Available from https://www.iaea.org/resources/rpop/health-professionals/nuclear-medicine/diagnostic-nuclear-medicine/misadministrations. [Accessed December 19, 2024].

[11] European Commission. Criteria for Acceptability of Medical Radiological Equipment used in Diagnostic Radiology, Nuclear Medicine and Radiotherapy. Radiation Protection n° 162; 2012.

[12] International Electrotechnical Commission. Nuclear medicine instrumentation – Routine tests – Part 4: Radionuclide calibrators. IEC TR 61948-4:2019, 2019. ISBN 978-2-8322-6602-1.

[13] Radiation Protection N 181 General guidelines on risk management in external beam radiotherapy; 2015.

[14] Huq M.S., Fraass B.A., Dunscombe P.B., Gibbons J.P., Ibbott G.S., Mundt A.J. (2016). The report of Task Group 100 of the AAPM: Application of risk analysis methods to radiation therapy quality management. Med Phys.

[15] Maughan N.M., Fraass B.A., Dunscombe P.B., Gibbons J.P., Ibbott G.S., Mundt A.J. (2016). The report of Task Group 100 of the AAPM: application of risk analysis methods to radiation therapy quality management. Med Phys.

[16] European Patent application no. EP 4 220 322 A1. https://worldwide.espacenet.com/publicationDetails/originalDocument?FT=D&date=20230802&DB=EPODOC&locale=en_EP&CC=EP&NR=4220322A1&KC=A1&ND=4.

[17] Kornek D., Menichelli D., Leske J., Hofmann M., Antkiewicz D., Brandt T. (2024). Development and clinical implementation of a digital system for risk assessments for radiation therapy. Z Med Phys.

[18] Younge K.C., Lee C., Moran J.M., Feng M., Novelli P., Prisciandaro J.I. (2016). Failure mode and effects analysis in a dual-product microsphere brachytherapy environment. Pract Radiat Oncol.

[19] Zoberi J.E., Garcia-Ramirez J., Luechtefeld D., Maughan N.M., Amurao M., Oyama R. (2023). Logistical, technical, and radiation safety aspects of establishing a radiopharmaceutical therapy program: a case in Lutetium-177 prostate-specific membrane antigen (PSMA) therapy. J Appl Clin Med Phys.

[20] Martin C.J., Marengo M., Vassileva J., Giammarile F., Poli G.L., Marks P. (2019). Guidance on prevention of unintended and accidental radiation exposures in nuclear medicine. J Radiol Prot.

[21] Rodrigues M.D.S.B., Oliveira S.M., de Sá L.V. (2021). Development of a model for registration and notification of accidents and incidents in nuclear medicine. J Radiol Prot.

[22] Younge K.C., Lee C., Moran J.M., Feng M., Novelli P., Prisciandaro J.I. (2016). Failure mode and effects analysis in a dual-product microsphere brachytherapy environment. Pract Radiat Oncol.

[23] Busse N.C., Al-Ghazi M.S.A.L., Abi-Jaoudeh N., Alvarez D., Ayan A.S., Chen E. (2024). AAPM Medical Physics Practice Guideline 14.a: Yttrium-90 microsphere radioembolization. J Appl Clin Med Phys.

[24] Levillain H., Bagni O., Deroose C.M., Dieudonné A., Gnesin S., Grosser O.S. (2021). International recommendations for personalised selective internal radiation therapy of primary and metastatic liver diseases with yttrium-90 resin microspheres. Eur J Nucl Med Mol Imaging.

[25] Chiesa C., Sjogreen-Gleisner K., Walrand S., Strigari L., Flux G., Gear J. (2021). EANM dosimetry committee series on standard operational procedures: a unified methodology for 99mTc-MAA pre- and 90Y peri-therapy dosimetry in liver radioembolization with 90Y microspheres. EJNMMI Phys.

