# Supplementary material for: Advancing risk management in nuclear medicine diagnostic and therapy through incident-driven risk management tools
Source: Z Med Phys. 2025 May 20;35(4):416–22. doi: 10.1016/j.zemedi.2025.03.004 (PMC12766496; doi:10.1016/j.zemedi.2025.03.004)
Supplement: Supplementary Data 1 — Failure modes for specific diagnostic and therapeutic scenarios considered in the paper. [file mmc1.docx]

**Table S1: Questionnaire – Diagnostic scenario**

Professional (Physician/Nurse/Medical Physicist/Secretary): …………………………………..

Email: ………………………………………………..

□ IRCCS AOU Bologna

□ AOU Novara

No. patients/year: ……………………………………..

Select one of the following radiopharmaceutical:

- ^18^F-FDG
- ^68^Ga-somatostatin analogues PET/^18^F-DOPA PET
- other

| Event | Expected cases  (N. pz/y) | Severity | Frequency | No. of detected events/100 events | Type of risk cause  O/P/TE/TR/I (*) |
| --- | --- | --- | --- | --- | --- |
| Exam booking (e.g. lack of information on comorbidities or drug allergies) |  |  |  |  |  |
| Failure in radiopharmaceutical labelling (e.g. QC not carried out due to extra workload, QC device problems, expired radiopharmaceutical kit, problems with the generator ...) |  |  |  |  |  |
| Error in vial measurement (e.g. miscalibration of dose calibrator) |  |  |  |  |  |
| Syringe preparation for the individual patient (incorrect identification of the patient, incorrect choice of radiopharmaceutical, ...) |  |  |  |  |  |
| Administration of inappropriate activity |  |  |  |  |  |
| Failure in patient preparation |  |  |  |  |  |
| Administration different from the planned one (extravasation or with different position of the catheter) |  |  |  |  |  |
| Administration interruption for clinical reasons attributable to the patient, administration with timing not adapted to the patient’s clinical conditions (e.g. decrease in renal biomarkers values or hepatic function, etc.) |  |  |  |  |  |
| Patient emesis and contamination of the gamma camera with possible artifacts or failure to examine the patient |  |  |  |  |  |
| Problems in radiopharmaceutical internal/external transport |  |  |  |  |  |
| Radiopharmaceuticals administration to pregnant and/or breastfeeding patient |  |  |  |  |  |
| Other |  |  |  |  |  |

* Abbreviations: O= Organization; P= Procedural; TE= Technical; TR=Training; I=Informatics

**Table S2: Questionnaire – Therapeutic scenario**

Professional (Physician/Nurse/Medical Physicist/Secretary): …………………………………..

Email: ………………………………………………..

□ IRCCS AOU Bologna

□ AOU Novara

No. patients/year: ……………………………………..

Select one of the following radiopharmaceutical:

- ^177^Lu-DOTA-peptide /^177^Lu-PSMA
- ^90^Y resion or glass microspheres /^166^Ho-loaded poly(L-lactic acid) microspheres
- Na^131^I
- other

| Event | Expected cases  (N. pz/y) | Severity | Frequency | No. of detected events/100 events | Type of risk cause  O/P/TE/TR/I (*) |
| --- | --- | --- | --- | --- | --- |
| First visit to select the appropriate therapy (e.g. lack of information on comorbidities or drug allergies) |  |  |  |  |  |
| Measurement of the vial containing the activity (e.g. uncalibrated calibrator) |  |  |  |  |  |
| Administration of the appropriate activity (e.g. incorrect LSF calculation or error in the activity calculation or treatment planning system) |  |  |  |  |  |
| Failure in patient preparation (antiemetic infusion, …) |  |  |  |  |  |
| Administration different from the planned one (extravasation or with different position of the catheter) |  |  |  |  |  |
| Administration interruption for clinical reasons attributable to the patient, administration with timing not adapted to the patient’s clinical conditions (e.g. decrease in renal biomarkers values or hepatic function, etc.) |  |  |  |  |  |
| Radiopharmaceuticals administration to pregnant and/or breastfeeding patient problems |  |  |  |  |  |
| Patient emesis and contamination of the gamma camera with possible artifacts or failure to examine the patient |  |  |  |  |  |
| Other |  |  |  |  |  |

* Abbreviations: O= Organization; P= Procedural; TE= Technical; TR=Training; I=Informatics
