# Supplementary material for: Advancing risk management in nuclear medicine diagnostic and therapy through incident-driven risk management tools
Source: Z Med Phys. 2025 May 20;35(4):416–22. doi: 10.1016/j.zemedi.2025.03.004 (PMC12766496; doi:10.1016/j.zemedi.2025.03.004)
Supplement: Supplementary Data 2 [file mmc2.docx]

**Table S3:** Failure modes for the seven steps of diagnostic scenarios according to the initial Severity (S_in_), Occurrence (O_in_), Detectability (D_in_), RPN_in_

| Step name | Cause of failure | Failure Mode | Initial Preventions | Initial Barriers | S_in_ | O_in_ | D_in_ | RPN_in_ | Effect |
| --- | --- | --- | --- | --- | --- | --- | --- | --- | --- |
| Patient admittance/booking | Wrong anamnesis | Pregnancy misidentification | Generic Prevention | Generic Barrier | 5 | 1 | 1 | 5 | PregMisIden |
| Patient admittance | Missing anamnesis | Breastfeeding misidentification | Generic Prevention | Generic Barrier | 3 | 1 | 1 | 3 | BreastFeed |
| Activity Preparation | Diagnostic-The wrong patient name has been identified | D-WrongPatient | Syringe labelling | Generic Barrier | 3 | 2 | 2 | 12 | WPN |
| Activity Preparation | Diagnostic-The wrong RF has been withdrawn | D-WrongRF | Generic Prevention | Generic Barrier | 1 | 1 | 4 | 4 | WRF |
| Activity Preparation | The wrong patient has been identified | T-WrongPatient | Generic Prevention | Generic Barrier | 10 | 2 | 1 | 20 | WPN |
| RF administration | Bad | T-RF administration | Generic Prevention | Generic Barrier | 10 | 1 | 2 | 20 | Extr |
| RF administration | RF extravasation while injecting | D-RF Extravasation | Generic Prevention | Generic Barrier | 3 | 1 | 1 | 3 | Extr |
| Image acquisition | Wrong acq protocol selected | T-Wrong acq protocol | Generic Prevention | Generic Barrier | 10 | 1 | 1 | 10 | T-Wrong protocol |
| Image acquisition | Missing pt infos, missing worker formation | D-Wrong acq protocol | Generic Prevention | Generic Barrier | 1 | 1 | 2 | 2 | D-Wrong protocol |
| Image processing | Therapy/The wrong protocol has been selected | T-Wrong proc protocol | Generic Prevention | Generic Barrier | 1 | 1 | 1 | 1 | T-Wrong protocol |
| Image processing | The wrong protocol has been selected | D-Wrong proc protocol | Generic Prevention | Generic Barrier | 1 | 2 | 2 | 4 | D-Wrong protocol |
| Patient dismission | Therapy/The patient has been dismissed earlier than planned - no medication provided | Earlier dismission | Generic Prevention | Generic Barrier | 5 | 1 | 1 | 5 | T-Earlier dismission |
| Archiving/reporting | Exam images not sent to PACS | Image archiving missing | Generic Prevention | Generic Barrier | 1 | 1 | 1 | 1 | NoImage |

**Table S4:** Failure modes for therapeutic NM scenarios according to the initial Severity (S_in_), Occurrence (O_in_), Detectability (D_in_), RPN_in_

| Step name | Substep name | Cause of failure | Failure Mode | Initial Preventions | Initial Barriers | S_in_ | O_in_ | D_in_ | RPN_in_ | Effect | Comments |
| --- | --- | --- | --- | --- | --- | --- | --- | --- | --- | --- | --- |
| Patient admittance | Start of patient evaluation | Lack of attention; time pressure | Blood draw mix-up | Secondary check of patient identity | (none) | 4 | 5 | 10 | 200 | No-harm | Adapted from ref. S1 Table 4.1 |
| Patient admittance | Collection and evaluation of clinical data (thyroid) | Lack of training; lack of experience | Incorrect thyroid volume determination with ultrasounds | Secondary physician check; staff training | Detected by chance | 2 | 7 | 8 | 112 | Inconvenience | Adapted from ref. S1 Table 4.1 |
| Pre-treatment planning | Verification of patient ID | Insufficient data (e.g. photo) available to recognize the patient; staff problem; heavy clinical workload | Patient mix-up | Verify patient demographic info, ask confirmation of patient name, use patient photo | Independent check by other staff | 10 | 2 | 2 | 40 | Mortality risk | Adapted from EBRT template |
| Pre-treatment planning | Imaging | Unknown or undocumented immune response to RP | Anaphylaxis | Check patient history, info for prevoius processes, anaphylaxis control. Ask patient/patient companion about allergies. | First aid availability | 9 | 2 | 5 | 90 | Permanent adverse effect | Adapted from EBRT |
| Pre-treatment planning | Imaging | Poor puncture technique; movement of patient | Extravasation | Staff training; patient relaxation | (none) | 3 | 4 | 10 | 120 | No-harm | Adapted from EBRT |
| Pre-treatment planning | Imaging | Poor image quality; lack of attention; time pressure | Misdiagnosis or inadequate targeting | Independent check by other staff member | Regular image reviews; checklist | 9 | 6 | 6 | 324 | Permanent adverse effect |  |
| Pre-treatment planning | Dosimetric evaluation with images | Lack of information; lack of experience; lack of attention | Incorrect calculation of activity | Independent check by other staff member | Detection by chance | 3 | 6 | 8 | 144 | No-harm | Adapted from ref. S1 Table 4.2 |
| Pre-treatment planning | Dosimetric evaluation with images | Insufficient information from images; insufficient image quality; lack of experience; time pressure | Incorrect determination of volumes based on images | Independent check by other staff member | Detected by chance | 3 | 6 | 8 | 144 | No-harm | Adapted from ref. S1 Table 4.2 |
| Pre-treatment planning | Dosimetric evaluation with images | Lack of attention; time pressure; lack of experience | Tc99 kit mix-up (liver tumors) | Independent check, review of kit | Detected by chance | 3 | 5 | 7 | 105 | No-harm | Adapted from ref. S1 Table 4.2 |
| Preparation of radiopharmaceutical | RP acceptance | Lack of attention to documents during transportation | Drug certificate lost | Training | Checked during subsequent workflow steps | 5 | 5 | 2 | 50 | Temporary adverse effect |  |
| Administration of radiopharmaceutical | Start of administration process | Lack of attention: lack of training | Patient does not following medical staff instructions | Attach additional information signs to room doors | Detected by change | 1 | 6 | 10 | 60 | Inconvenience | Adapted from ref. S1 Table 4.1 |
| Administration of radiopharmaceutical | Capsule application (Iodine) | Inventory not up to date; lack of attention | Capsules mix-up | Train staff to work with attention | Detected by chance | 4 | 6 | 2 | 48 | No-harm | Adapted from ref. S1 Table 4.1 |
| Administration of radiopharmaceutical | Liquid application | Lack of attention; lack of information; insufficient questioning of patient | Unintentional swap of administration body side (left/right) | Independent check by other staff member | Detected by chance | 5 | 4 | 5 | 100 | Temporary adverse effect | Adapted from ref. S1 Table 4.3 |
| Administration of radiopharmaceutical | Liquid application | Lack of attention; lack of information; insufficient information flow to physician/radiologist | Incorrect placement of catheter | Independent check by other staff member | Detected by chance | 6 | 4 | 3 | 72 | Temporary adverse effect | Adapted from ref. S1 Table 4.2 |
| Administration of radiopharmaceutical | Liquid application | Lack of experience; patient mobility | Misplacing of injection cannula | Independent check by other staff member; periodic staff training | Detected by chance | 5 | 5 | 5 | 125 | Temporary adverse effect | Adapted from ref. S1 Table 4.3 |
| Administration of radiopharmaceutical | Device application | Lack of attention; lack of experience; time pressure | Vial with incorrect activity handled to the physician | Consultation between doctor and nursing staff | Detected by chance | 5 | 4 | 7 | 140 | Temporary adverse effect | Adapted from ref. S1 Table 4.3 |
| Administration of radiopharmaceutical | Device application | Incorrect application of procedures; lack of experience; lack of attention | Embolization | Independent check by other staff member; flux evaluation by radiologist | Detected by chance | 6 | 2 | 5 | 60 | Temporary adverse effect | Adapted from ref. S1 Table 4.2 |
| Administration of radiopharmaceutical | Liquid application | Lack of attention | Reverse flow of activity (back flow) | Independent check by other staff member | Detected by chance. | 6 | 5 | 6 | 180 | Temporary adverse effect | Adapted from ref. S1 Table 4.3 |
| Administration of radiopharmaceutical | Liquid application | Lack of attention; lack of experience; patient movement during process | Incorrect pull out of catheter | Checks bandage pressure | Detected by chance | 6 | 2 | 3 | 36 | Temporary adverse effect | Adapted from ref. S1 Table 4.2 |
| Patient follow-up | Collect patient feedback | Lack of attention | Patient forget to call back during follow up | Patient education | Detected by chance | 1 | 7 | 8 | 56 | Inconvenience | Adapted from ref. S1 Table 4.3 |

References

S1 Kotzerke J, Fetzer P, Grosche-Schlee S, Hanel A, Freudenberg R, Brogsitter C. Entwicklung eines Systems zur Risikoanalyse bei der Behandlung mit offenen radioaktiven Stoffen in der Nuklearmedizin [Development of a system for risk analysis in treatment with unsealed radioactive substances in nuclear medicine]. Nuklearmedizin. 2020 Apr;59(2):60-78. German. doi: 10.1055/a-1119-0849. Epub 2020 Mar 3. PMID: 32126578.
